# Carbon footprint of self-selected US diets: nutritional, demographic, and behavioral correlates

**DOI:** 10.1093/ajcn/nqy327

**Published:** 2019-01-29

**Authors:** Donald Rose, Martin C Heller, Amelia M Willits-Smith, Robert J Meyer

**Affiliations:** 1 Tulane University, School of Public Health and Tropical Medicine, New Orleans, LA; 2 University of Michigan, Center for Sustainable Systems, School for Environment and Sustainability, Ann Arbor, MI

## Abstract

**Background:**

A substantial portion of greenhouse gas emissions (GHGE) has been attributed to the food sector, but little is known about the association between the carbon footprint of individual self-selected diets in the United States and nutritional quality.

**Objectives:**

The aims of this study were to assess the GHGE from individual self-selected diets in the United States and examine their association with nutritional quality of the diets, demographic patterns, and food-related behaviors.

**Methods:**

The dietary GHGE from US adults (>18 y, *N* = 16,800) in the 2005–2010 National Health and Nutrition Examination Survey (NHANES) were calculated by linking all foods consumed in their 24-h recall diets to our new database of food environmental impacts. Diets were ranked by GHGE/1000 kcal. Those in the top and bottom quintiles were compared on the US Healthy Eating Index (HEI) and on the amounts of specific nutrients known to be under- or overconsumed in the US population. Demographic and behavioral variables from the NHANES were also correlated to these dietary carbon footprints.

**Results:**

Diets in the bottom quintile accounted for one-fifth the total emissions (GHGE/1000 kcal) of those in the top quintile, yet had significantly higher (*P* < 0.001) HEI scores by 2.3 ± 0.7 points on a 100-point scale. These low-GHGE diets contained higher amounts of fiber and vitamin E and lower amounts of sodium and saturated fats, whereas high-GHGE diets contained higher amounts of vitamins A and D, choline, calcium, iron, and potassium. Low-GHGE diets had less meat, dairy, and solid fats, and more poultry, plant protein foods, oils, whole and refined grains, and added sugars.

**Conclusions:**

Food patterns responsible for lower GHGE had a better overall diet quality and were more nutritious on several key dimensions, although not all. These results can inform dietary guidance and other policies that seek to address the goals of improved dietary intakes and reduced food-related emissions.

## Introduction

Global climate change is arguably the most urgent environmental problem faced today. According to global annual temperature records, which date back to 1880, 17 of the 18 hottest years have occurred in the 21st century (1, 2). Correspondingly, the incidence of daily tidal flooding due to global sea level rise is accelerating in more than 25 Atlantic and Gulf Coast cities (3). The negative health effects of climate change around the world are unequivocal (4). Human-induced climate change threatens to undermine the gains in public health made over the last 50 y (5).

Food production is one of the largest contributors to climate change. Much of the impact comes from the types of foods produced, which is influenced by consumer demand. The latest estimates from the UN FAO reveal that the greenhouse gas emissions (GHGE) associated with the production of just meat and dairy account for 14.5% of the global total (6). Based on a systematic review, Hallström et al. (7) have estimated that by changing current diets, GHGE from food could be reduced by up to 50%.

The most recent US Dietary Guidelines Advisory Committee claimed that shifts toward more plant-based foods could promote health as well as long-term environmental sustainability of the nation's food supply (8). Evidence used to reach this conclusion came primarily from studies outside the United States, since little is known about how individual food choices in this country are jointly related to both environmental impacts and nutritional quality. In fact, most studies of diet-environment-nutrition linkages have been conducted on aggregate national averages, stereotypical diets (e.g., vegetarian), or on diets optimized by investigators (9–14). Although there is growing evidence that healthful diets could reduce environmental impacts, with some exceptions (15–18) the linkage has not been adequately studied at the individual level. This makes it difficult to estimate the relationship between the environmental impacts of self-selected diets and their healthiness. It also precludes prediction of the variation of impacts of a given policy choice, such as revised dietary guidance or food labeling, or to know where to target potential interventions.

To address this gap in the literature, our research has 2 main objectives: *1*) to develop and implement a method for linking food environmental impact data to a large nationally representative diet survey of the US population; and *2*) to describe the nutritional, demographic, and behavioral correlates of GHGE from the production of food (i.e., the carbon footprint) of US diets. We relied on an interdisciplinary effort between nutritionists and environmental scientists to develop a distribution of carbon footprints for US diets (19). We then compared diets in the bottom and top quintiles of this distribution on a number of dimensions. Our primary outcome variables are nutrients and food components of public health concern. Our secondary outcome variables are demographic and behavioral correlates of dietary GHGE.

## Methods

### Study sample

The study sample used for this analysis came from the National Health and Nutrition Examination Survey (NHANES), a multistage, nationally representative survey of the US civilian, noninstitutionalized population. Since 1999, NHANES has been conducted on an ongoing basis in 2-y rounds. We employed the 3 rounds of data collection from 2005 to 2010. Our sample (*N* = 16,800) consisted of all adult individuals during these rounds, who were aged ≥18 y and who completed a reliable interviewer-administered 24-h dietary recall.

### Dietary recall data

Trained interviewers collected information on all food and drink consumed by individual respondents in the preceding 24 h (midnight to midnight) with the use of the USDA's Automated Multiple Pass Method for Dietary Recalls (20). Interviews were conducted in the NHANES Mobile Examination Center and included items to help elicit portion sizes from respondents, such as glasses, bowls, measuring spoons, and images.

The USDA is responsible for this dietary data collection methodology as well as the review and processing of data in the dietary component of NHANES, also known as What We Eat in America. The overall acceptability of each recall was determined by USDA according to a protocol that incorporated information missing from the recall as well as from postinterview remarks by the interviewer (21). Only those recalls that were deemed reliable and that met the minimum criteria for acceptability were included in our analysis. Extensive documentation of these NHANES dietary procedures has been published previously (22).

### Environmental impacts and linkage to dietary data

An extensive review of the life cycle assessment (LCA) literature was conducted to develop a database of environmental impacts of foods, specifically GHGE and cumulative energy demand. Food LCA studies examine the relevant stages of a supply chain for a specific food, modeling the inputs and outputs at each stage and the associated environmental impacts (23). We recorded information from the LCA literature for all relevant publications from 2005 to 2016 into a database of Food Impacts on the Environment for Linking to Diets (dataFIELD). Values of GHGE were recorded in kg CO_2_-equivalents (CO_2_-eq) per kg of commodity. This standard unit is used to put various gases, including carbon dioxide, methane, and nitrous oxide, on the same scale with respect to their global warming potential. For commodities with several studies in the literature, we took a simple average and used this for linking to dietary data. For commodities in which there were no LCA studies, we used the data from a group of foods (e.g., stone fruits for nectarines), or a substitute food of similar form (e.g., banana for plantain). Because the vast majority of studies only considered emissions up to the farm gate, our GHGE factors captured only the production, and in some cases primary processing, of foods. Extensive details about the development of this database have been published previously (19) and the databases will be made available at http://css.umich.edu/page/datafield and at https://sph.tulane.edu/gchb/diet-environmental-impacts.

**FIGURE 1:**
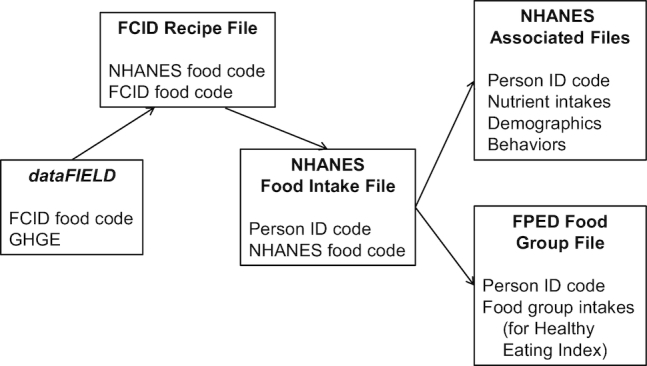
Schematic of how environmental impact data were linked to food, nutrient, demographic, and behavioral variables. dataFIELD, database of Food Impacts on the Environment for Linking to Diets; FCID, Food Commodity Intake Database; FPED, Food Pattern Equivalent Database; GHGE, greenhouse gas emissions.

The linkage of greenhouse gas emissions recorded in dataFIELD to most foods in NHANES diets was accomplished through the use of food codes in the Food Commodity Intake Database (FCID) (24). FCID was developed by the US Environmental Protection Agency to assist efforts in understanding potential pesticide exposures from food of the US population. Individual food items as eaten and reported in the NHANES dietary recalls were translated into commodity form through thousands of recipes. For the 2005–2010 waves, there were 11,658 NHANES items with recipes that were composed of 332 different FCID commodity ingredients. Each recipe listed the gram quantities of all the FCID ingredients needed to make up 100 g of an NHANES food. For example, an FCID recipe for a pepperoni pizza consumed by an NHANES respondent consisted of the commodity ingredients of wheat flour, tomato, milk, pork, onion, etc. GHGE values (CO_2_-eq/kg) for each of these commodity ingredients came from dataFIELD. We summed the GHGE of all the ingredients, adjusting for recipe quantities and amounts eaten, to derive the GHGE for each food or dish reported by the NHANES respondents. See **Figure 1** for a schematic of the linkages of the various databases.

In certain instances, we linked environmental variables directly to NHANES foods. A number of LCA studies have been conducted on processed foods, e.g., cheese, tofu, or sodas. In these cases we connected GHGE values for these foods directly to the NHANES items. For mixed drink cocktails and other liquors we developed our own recipe file and used that to link to NHANES foods.

Once all foods consumed by individuals had environmental impacts associated with them, we aggregated these impacts for each individual, adding up GHGE for all foods consumed on the interview day. We included impacts associated with the amount of each food consumed as well as estimated losses both at the retail and consumer level. Estimates of losses at these levels were obtained from the Loss-Adjusted Food Availability dataset, produced by USDA (25).

### Dietary analysis variables

Specific nutrients or food components examined were those listed as “nutrients of concern” in the 2015–2020 Dietary Guidelines for Americans (DGA), because they are either under- or overconsumed by segments of the population and may pose a substantial public health problem (26). Underconsumed nutrients included iron, magnesium, calcium, potassium, fiber, choline, and vitamins A, C, D, and E. Overconsumed components included sodium and saturated fats. Because of our overriding interest in the composition of diet, rather than total amounts consumed, all nutrient quantities were reported as densities, i.e., per 1000 kcal.

Quantities of individual foods reported in NHANES were converted to food groups according to the Food Pattern Equivalent Database, which is developed by USDA in 2-y cycles that match the NHANES cycles, in this case for 2005–06, 2007–08, and 2009–10 (27). In order to put dried, cooked, and fresh foods on an equivalent scale and assist in consumer guidance and monitoring, Food Pattern Equivalent Database foods were reported in cup-equivalents (fruit, vegetables, and dairy), ounce equivalents (grains, protein foods), teaspoon equivalents (added sugars), or gram equivalents (solid fats and oils). As with nutrients, all quantities were scaled per 1000 kcal.

Since it is difficult to draw conclusions about the overall healthiness of diets by evaluating individual nutrients or foods, we also examined a summary measure of dietary quality, the Healthy Eating Index (HEI). The HEI was originally developed by USDA's Center for Nutrition Policy and Promotion in 1995 (28), and has been updated 3 times since then to be consistent with the latest dietary advice in the DGA (29, 30). The 2010 version of the HEI was divided into 12 components, the maximum scores of which sum to 100 points (30). Nine of these components reflected aspects of the diet that should be encouraged, including whole fruits (5 points), total fruits (5 points), greens and beans (5 points), total vegetables (5 points), whole grains (10 points), dairy (10 points), total protein foods (5 points), seafood and plant proteins (5 points), and fatty acids (10 points). Three components reflected aspects of the diet that should be reduced, including refined grains (10 points), sodium (10 points), and empty calories (20 points) (30). The fatty acids component score was based on a ratio of poly- plus monounsaturated fatty acids divided by saturated fatty acids. The empty calories component included solid fats, alcohol, and added sugars. HEI component scores were calculated at the individual level with the use of previously defined algorithms (31). For the fatty acids and sodium components, scores were based on data from the NHANES nutrients file.

### Demographic, socioeconomic, and behavioral variables

We linked the GHGE from individuals’ diets with their demographic, socioeconomic, and behavioral variables obtained from other NHANES modules. Demographic variables, including age, gender, race-ethnicity, education, and income, came from the NHANES demographic survey module. Age was categorized into 4 groups (18–29, 30–49, 50–65, and ≥66 y). NHANES age data were top-coded at 80 y. Race-ethnicity was self-reported on NHANES and this was recoded into 4 groups: Latino (“Mexican-American” and “Other Hispanic”), White (“Non-Hispanic White”), African-American (“Non-Hispanic Black”), and “Other”, which includes individuals who identified as multiracial. Education was also categorized into 4 groups: less than a high-school diploma; high-school graduate or equivalent; some college; and college graduate. There were 5 income groups that were based on multiples of the income-to-poverty ratio (i.e., the ratio of family income to the federal poverty guideline for each family, based on its family size, state of residence, and year of interview). These income groups were: less than the poverty guideline, 1–2 times, 2–5 times, and >5 times the poverty guideline. An additional group was added for those who did not report their income.

Knowledge and behavioral variables came from either the Diet Behavior and Nutrition Module or the Consumer Behavior Phone Follow-up Module for Adults, the latter of which was only fielded from 2007–2010. *Use of food labels* was a dichotomous variable created to indicate that the respondent used some component of the food label at least sometimes. This was developed from 4 other variables on the module, indicating use of the nutrition facts panel, the ingredients list, the serving size, or a health claim. Those who responded that they sometimes, most of the time, or always used any 1 of these 4 label components were defined as using food labels. Those who rarely or never used any of these components were defined as not using food labels. *Heard of dietary guidance* was another knowledge dichotomous indicator that combined information on whether the respondent had heard of the Food Guide Pyramid (in 2005–06) or had heard of either the Food Guide Pyramid or MyPyramid (in 2007–10). Both the variables *use of food labels* and *heard of dietary guidance* were available on all respondents in our sample.

Data for the variables *used dietary guidance, main meal preparer*, and *self-perceived vegetarian* were only available for 2007–10, because questions on NHANES behavioral modules vary from wave to wave. Those who tried to follow a MyPyramid plan were defined as having *used dietary guidance*. Respondents reporting that they were the ones who did most of the planning or preparing of meals in their families were the *main meal preparers*, also a dichotomous variable. Respondents were asked if they considered themselves to be a vegetarian. Those answering “yes” were defined as *self-perceived vegetarians*.

### Statistical analyses

All analyses were conducted in Stata/SE 13.1 (StataCorp) and made use of the statistical weights and sampling design parameters of the NHANES (32). In order to focus on food choices independent of energy requirements, individual diets were ranked according to GHGE per 1000 kcal. Those in the first (lowest) and fifth (highest) quintile groups were compared on the variables described above. Throughout the paper we refer to these as either the low- or high-GHGE diets, or the low- or high-carbon footprint diets. Chi-square statistics were used to detect demographic differences in **Table 1**. *t* tests were used to detect differences in **Tables 2–4**. For **Table 5**, ordinary least-squares regression was used to assess the independent effect of behaviors on dietary GHGE after controlling for basic demographic and socioeconomic variables described above. An α level of 0.05 was used to determine statistical significance. For **Supplemental Tables 1–3**, linear regression was used to assess differences in dietary components between all 5 quintile groups, with the first quintile as the reference group. Linear and quadratic trends between the 5 quintiles were also tested.

**TABLE 1:** Demographic characteristics of the study sample and of those consuming low- and high-GHGE diets, adults ≥18 y, NHANES 2005–2010^1^

	Overall sample (*n* = 16,800)	Low-GHGE diet (*n* = 3545)	High-GHGE diet (*n* = 3303)	*P* ^2^
Gender	—	—	—	<0.001
Female, %	52.1	56.1	46.5	
Male, %	47.9	43.9	53.5	
Age, y	—	—	—	0.010
18–29, %	22.1	25.8	21.1	
30–49, %	36.9	36.3	37.4	
50–65, %	25.5	24.0	25.8	
≥66, %	15.5	14.0	15.7	
Race-ethnicity	—	—	—	<0.001
Latino, %	12.7	13.2	12.5	
White, %	70.1	66.6	71.1	
African-American, %	11.6	14.3	10.3	
Other, multi, %	5.7	6.0	6.1	
Education	—	—	—	0.352
<High school graduate, %	19.2	20.4	19.6	
High school graduate, %	25.0	24.8	26.2	
Some college, %	30.6	31.4	29.6	
College graduate, %	25.2	23.4	24.6	
Income-to-poverty ratio^3^	—	—	—	0.754
Missing income data, %	6.2	6.6	5.8	
<1, %	13.2	14.4	14.2	
1–<2, %	19.1	19.5	19.4	
2–<5, %	37.0	36.7	36.4	
≥5, %	24.4	22.7	24.2	

1 Diets in NHANES were ranked on GHGE (kg CO_2_-eq/1000 kcal per day), and divided into quintiles. Those in the lowest quintile of GHGE were defined as low-GHGE diets, whereas those in the top quintile were defined as high-GHGE diets. GHGE, greenhouse gas emissions.

2 Determined by chi-square test.

3 Income-to-poverty ratio is the ratio of family income to the federal poverty guideline for each family based on its household size, state of residence, and the year of observation. An income-to-poverty ratio <1 indicates the family is in poverty.

## Results

**FIGURE 2:**
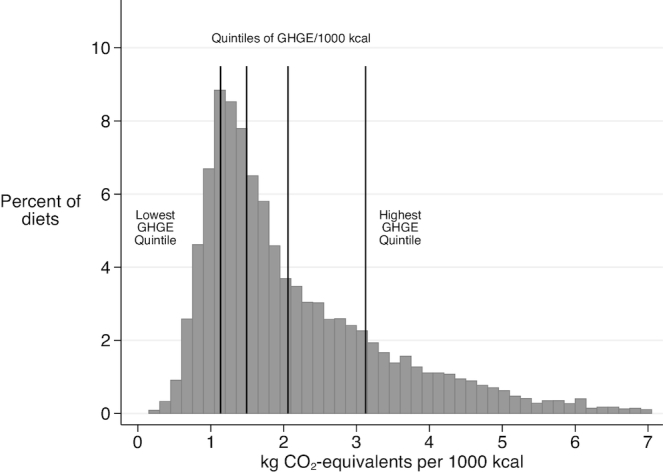
Distribution of dietary GHGE from 1-d diets, NHANES 2005–2010, kg CO_2_-eq/1000 kcal. CO_2_-eq, carbon dioxide equivalent; GHGE, greenhouse gas emissions.

The average dietary GHGE of this US sample, including both consumed food and food losses, was 4.72 kg CO_2_-eq per person per day (95% CI: 4.62, 4.82) and 2.21 kg CO_2_-eq per person per 1000 kcal (95% CI: 2.17, 2.24). The frequency distribution of GHGE per 1000 kcal from these 1-d diets is shown in **Figure 2**. The sample was divided into quintile groups, and the cumulative GHGE from the lowest quintile group represented 8% of the total GHGE from diet, whereas for the top group it was 41%.

The demographic characteristics of the overall study sample are presented in Table 1 in the first data column. The sample was roughly split between females (52%) and males (48%), and ∼22% of the population was between 18 and 30 y of age. About a third of the population had an income less than 2 times the poverty guideline, and the race-ethnic composition of the population was similar to that of the United States, of which the sample was designed to be representative. Comparisons of the top and bottom quintile groups on demographics revealed significant differences with respect to age, gender, and race-ethnicity, but not in terms of education or income. Specifically, the low-emitting diets were more likely to be consumed by women, those <30 y, and African-Americans (Table 1, data columns 2 and 3).

The nutrient composition of the reported diets was examined for the low- and high-GHGE groups (Table 2). The dietary fiber content of the diets in the low-GHGE group averaged 8.8 g/1000 kcal, which was significantly higher (*P* < 0.001) than the mean of the high-GHGE group (7.1 g/1000 kcal). Low-GHGE diets also had significantly more vitamin E (4.0 compared with 3.4 mg/1000 kcal) and significantly less sodium (1510 compared with 1775 mg/1000 kcal) and saturated fats (9.9 compared with 13.5 g/1000 kcal). On the other hand, high-GHGE diets had a higher concentration of vitamin A, vitamin D, choline, iron, calcium, and potassium.

**TABLE 2:** Nutrient intakes per 1000 kcal in low- and high-GHGE diets, adults ≥18 y, NHANES 2005–2010^1^

	Low-GHGE diet^2^ (*n* = 3545)	High-GHGE diet^2^ (*n* = 3303)	*P* ^3^
GHGE, kg CO_2_ eq/1000 kcal	0.90 ± 0.00	4.54 ± 0.03	
% total GHGE/1000 kcal	8.2%	41.1%	
Dietary fiber, g/1000 kcal	8.77 ± 0.16	7.11 ± 0.11	<0.001
Vitamin A, µg RAE/1000 kcal	269.21 ± 7.97	304.80 ± 9.96	0.006
Vitamin C, mg/1000 kcal	40.10 ± 1.28	43.03 ± 1.52	0.145
Vitamin D (D_2_ + D_3_), µg/1000 kcal	1.75 ± 0.05	2.11 ± 0.06	<0.001
Vitamin E as α-tocopherol, mg/1000 kcal	3.99 ± 0.08	3.38 ± 0.1	<0.001
Total choline, mg/1000 kcal	119.72 ± 1.27	192.99 ± 1.81	<0.001
Iron, mg/1000 kcal	7.44 ± 0.11	7.99 ± 0.1	<0.001
Calcium, mg/1000 kcal	393.67 ± 5.24	457.34 ± 5.71	<0.001
Magnesium, mg/1000 kcal	145.21 ± 1.92	145.79 ± 1.6	0.787
Potassium, mg/1000 kcal	1165.89 ± 13.16	1421.55 ± 16.04	<0.001
Sodium, mg/1000 kcal	1510.42 ± 12.95	1774.93 ± 15.96	<0.001
Total saturated fatty acids, g/1000 kcal	9.85 ± 0.09	13.46 ± 0.09	<0.001

1 Values are mean ± SE. CO_2_-eq, carbon dioxide equivalent; GHGE, greenhouse gas emissions; RAE, retinol activity equivalent.

2 Low-GHGE diets are defined as those in the lowest quintile of GHGE (kg CO_2_-eq/1000 kcal per day). High-GHGE diets are defined as those in the highest quintile of GHGE per 1000 kcal per day.

3 Determined by *t* test. Statistical tests were not run on the GHGE continuous variable (first 2 rows), because the quintile groups were based on it.

The food composition of the low- and high-GHGE diets is reported in Table 3 . On average, the low-GHGE diets had more whole grains, refined grains, poultry, plant protein foods (legumes, soybeans, nuts, and seeds), oils, and added sugars per 1000 kcal than the high-GHGE diets. The high-GHGE diets had greater quantities of vegetables, meat (beef, veal, other ruminant animals, pork, and game), seafood, dairy, and solid fats per 1000 kcal than the low-GHGE diets. Overall, the high-GHGE diets were more concentrated in total protein foods and animal protein foods.

**TABLE 3:** Food group intakes by low- and high-dietary GHGE groups, adults ≥18 y, NHANES 2005–2010^1^

Food group	Unit^2^	Low-GHGE diet^3^ (*n* = 3545)	High-GHGE diet^3^ (*n* = 3303)	*P* ^4^
Total fruit and vegetables^5^	cup eq/1000 kcal	1.19 ± 0.03	1.30 ± 0.03	0.007
Fruit	cup eq/1000 kcal	0.49 ± 0.02	0.46 ± 0.02	0.255
Vegetables^5^	cup eq/1000 kcal	0.71 ± 0.02	0.84 ± 0.02	<0.001
Total grains	oz eq/1000 kcal	3.60 ± 0.05	2.63 ± 0.03	<0.001
Whole grains	oz eq/1000 kcal	0.51 ± 0.02	0.28 ± 0.01	<0.001
Refined grains	oz eq/1000 kcal	3.09 ± 0.05	2.35 ± 0.03	<0.001
Protein foods: total^6^	oz eq/1000 kcal	2.37 ± 0.04	4.17 ± 0.04	<0.001
Animal protein foods	oz eq/1000 kcal	1.57 ± 0.03	3.79 ± 0.04	<0.001
Meat^7^	oz eq/1000 kcal	0.11 ± 0.01	2.26 ± 0.04	<0.001
Poultry	oz eq/1000 kcal	0.85 ± 0.03	0.33 ± 0.02	<0.001
Seafood	oz eq/1000 kcal	0.21 ± 0.01	0.41 ± 0.03	<0.001
Plant protein foods^8^	oz eq/1000 kcal	0.81 ± 0.04	0.37 ± 0.02	<0.001
Total dairy	cup eq/1000 kcal	0.54 ± 0.01	0.72 ± 0.02	<0.001
Oils	g/1000 kcal	12.86 ± 0.23	8.40 ± 0.14	<0.001
Solid fats	g/1000 kcal	14.67 ± 0.23	19.02 ± 0.16	<0.001
Added sugars	tsp eq/1000 kcal	10.46 ± 0.25	7.43 ± 0.15	<0.001

1 Values are mean ± SE. CO_2_-eq, carbon dioxide equivalent; cup eq, cup equivalent; GHGE, greenhouse gas emissions; oz eq, ounce equivalents; tsp eq, teaspoon equivalents.

2 Units for food groups were developed by USDA in common units and on an equivalent basis to create nutritional homogeneity in groups that have foods with diverse water concentrations (e.g., juice, fruit, or dried fruit). Cup eq/1000 kcal refers to cup equivalents per 1000 kcal. For example, 1 cup equivalent of dairy is either 1 cup (245 g) of milk, yogurt, or fortified soy milk, ∼1.5 oz of natural cheese, or ∼2 oz of processed cheese. See (27) for additional details.

3 Low-GHGE diets are defined as those in the lowest quintile of GHGE (kg CO_2_-eq/1000 kcal per day). High-GHGE diets are defined as those in the highest quintile of GHGE per 1000 kcal per day.

4 Determined by *t* test.

5 Vegetable totals do not include legumes.

6 The total protein foods group is a sum of animal and plant protein foods.

^7^The meat group includes beef, veal, other ruminant animals, pork, and game.

8 The plant protein foods group includes all legumes, soybeans, nuts, and seeds.

Overall evaluation of food composition of these diets showed that total HEI scores for the low-GHGE diets were significantly higher than the high-GHGE diets (50.2 compared with 48.0). Low-GHGE diets scored significantly higher on the whole fruit component of the HEI, 2.1 compared with 1.8 out of a total of 5 points (Table 4). Low-GHGE diets also scored higher on the whole grains, seafood and plant proteins, fatty acids, and sodium components, whereas high-GHGE diets scored higher on the total vegetable, dairy, total protein foods, and refined grains components.

**TABLE 4:** HEI component and total scores by low- and high-dietary GHGE groups, adults ≥18 y in the 2005–10 NHANES^1^

HEI component	Maximum score	Low-GHGE diet^2^ (*n* = 3545)	High-GHGE diet^2^ (*n* = 3303)	*P* ^3^
Total fruit	5	2.04 ± 0.07	1.91 ± 0.05	0.134
Whole fruit	5	2.06 ± 0.08	1.77 ± 0.05	0.003
Total vegetables	5	2.80 ± 0.05	3.18 ± 0.05	<0.001
Greens and beans	5	1.14 ± 0.05	1.19 ± 0.06	0.510
Whole grains	10	2.74 ± 0.09	1.77 ± 0.07	<0.001
Dairy	10	4.03 ± 0.08	4.83 ± 0.10	<0.001
Total protein foods	5	3.56 ± 0.04	4.87 ± 0.01	<0.001
Seafood and plant proteins	5	2.38 ± 0.06	1.60 ± 0.06	<0.001
Fatty acids	10	6.91 ± 0.08	3.59 ± 0.07	<0.001
Refined grains^4^	10	5.22 ± 0.12	7.05 ± 0.08	<0.001
Sodium^4^	10	5.57 ± 0.09	3.99 ± 0.10	<0.001
Empty calories^4,5^	20	11.81 ± 0.23	12.26 ± 0.15	0.065
Total HEI score	100	50.25 ± 0.57	48.00 ± 0.42	<0.001

1 Values are mean ± SE. The HEI is an overall index of diet quality based on the Dietary Guidelines for Americans. The 2010 version was used for this analysis (30). CO_2_-eq, carbon dioxide equivalent; GHGE, greenhouse gas emissions; HEI, Healthy Eating Index.

2 Low-GHGE diets are defined as those in the lowest quintile of GHGE (kg CO_2_-eq/1000 kcal per day). High-GHGE diets are defined as those in the highest quintile of GHGE per 1000 kcal per day.

3 Determined by *t* test.

4 Higher component scores are considered beneficial. Thus, for refined grains, sodium, and empty calories, higher scores indicate diets that contain less of these items.

5 Calories from solid fats, added sugars, and alcohol. For alcohol, intakes ≤13 g/1000 kcal do not influence scoring.

Supplemental Tables 1–3 report mean nutrient intakes, food group intakes, and HEI component scores, respectively, for all 5 quintiles of the GHGE-diets. For many of the nutrients and food groups—such as fiber, vitamin E, sodium, saturated fats, meats, plant protein foods, oils, and whole grains—intakes monotonically decreased (or increased) between the lowest and highest GHGE quintile diets. However, many of the relationships were nonlinear. For example, the third GHGE quintile diets had intakes of vitamin A, calcium, and dairy foods that were higher than either the first- or fifth-quintile diets.

We examined behavioral correlates of dietary GHGE. On average, self-perceived vegetarians consumed diets with significantly lower GHGE, by 0.80 kg CO_2_-eq/1000 kcal, than nonvegetarians (Table 5). Those who had used food labels, had heard of dietary guidance, or had tried dietary guidance also consumed diets with a lower carbon footprint. This was also true for the main meal preparers in the family. Most of these associations were still significant after controlling for age, gender, race-ethnicity, income, and education (Table 5, data columns 3–6). Only the main meal preparer result was no longer significant after controlling for these variables, whereas “having heard of dietary guidance” was only marginally significant in the full model (Table 5, data columns 3–6).

**TABLE 5:** Relationship between dietary GHGE per 1000 kcal and behavioral variables reported in the 2005–10 NHANES, adults ≥18 y^1^

	Unadjusted models^2^		Models controlling for demographic variables^2^		Models controlling for demographic and socioeconomic variables^2^	
Behavior	Coef (SE)^3^	*P* ^4^	Coef (SE)^3^	*P* ^4^	Coef (SE)^3^	*P* ^4^
Used food labels	–0.166 (0.048)	0.001	–0.136 (0.048)	0.007	–0.125 (0.048)	0.012
Heard of dietary guidance	–0.134 (0.043)	0.003	–0.104 (0.048)	0.035	–0.087 (0.045)	0.062
Tried dietary guidance	–0.129 (0.039)	0.002	–0.095 (0.038)	0.019	–0.081 (0.038)	0.041
Main meal preparer	–0.070 (0.029)	0.020	–0.009 (0.034)	0.794	–0.006 (0.035)	0.859
Self-perceived vegetarian	–0.802 (0.076)	<0.001	–0.775 (0.070)	<0.001	–0.766 (0.069)	<0.001

1 GHGE, greenhouse gas emissions.

2 The dependent variable in all models is GHGE (kg CO_2_-eq/1000 kcal). Each row represents a separate set of models. For unadjusted models, the dietary GHGE is regressed solely on the behavior. Models controlling for demographic variables included age, gender, and race-ethnicity. The final model set included these variables plus income category and educational level.

3 Coef is the β coefficient in each of these models and represents the mean difference in dietary GHGE (kg CO_2_-eq/1000 kcal) between those with the behavior and those without it. For example, in the unadjusted model, individuals who used food labels had a mean dietary GHGE that was lower than those who did not use labels by 0.166 kg CO_2_-eq/1000 kcal.

4 Determined by *t* test.

## Discussion

We found a tremendous variation in the size of carbon footprints of 1-d diets from a nationally representative sample of US adults. Ranked in ascending order of GHGE per 1000 kcal, we found the highest quintile diets contributed 41% of total dietary emissions (GHGE/1000 kcal), 5.1 times the 8% of emissions from the lowest quintile. There were significant differences in the nutrient composition of these low- and high-carbon footprint diets. The low-GHGE diets contained more fiber and vitamin E, and less sodium and saturated fats. But the nutrient profiles of these diets were not superior on all dimensions, since they contained significantly less iron, calcium, vitamin D, vitamin A, choline, and potassium than the high-GHGE diets. This is likely due to the lower consumption of animal foods, meats, and dairy, which was also seen in the HEI subcomponent scores. The HEI provides an authoritative, overall measure of the healthiness of a given diet, one that has been validated previously (33). Despite the fact that the low-GHGE diets scored worse on some components, including protein foods, dairy, vegetables, and refined grains, they scored significantly better on others, including whole fruit, whole grains, seafood and plant proteins, fatty acids, and sodium. On average, total HEI scores for the low-GHGE diets were significantly higher than for the high-GHGE diets.

This inverse relationship between environmental impact and healthiness of the diet is consistent with a number of different types of studies in various settings. In the Netherlands, improvements in an overall health score of Dutch diets were positively correlated with improvements in a sustainability score based on GHGE and land use (13). In the United States, based on the use of aggregate national consumption averages, Heller and Keoleian (9) found that moving to a diet based on some of the patterns in the DGA (i.e., vegetarian or vegan) would result in significant reductions in GHGE. Hallström et al. (34) also used aggregate data to show that a diet based on recommendations would lower GHGE from the average American diet. A significant reduction in environmental impacts could be obtained if consumers followed either of 2 sets of healthy dietary recommendations developed for Germany (35). Other researchers, studying diets in the United Kingdom (12), France, Spain, Sweden (36), and New Zealand (11), have used mathematical optimization techniques to show that diets can be developed that would improve both health and sustainability over current diets. These findings have also been seen on a global level (37).

Not all studies have documented this inverse relationship between environmental impact and healthiness of the diet. Tom et al. (38) found that a potential shift from the current average US diet to one recommended in the 2010 DGA would increase GHGE. Vieux et al. (15), studying self-selected French diets, found that less impactful diets were associated with lower nutritional quality. Despite differences between our studies in overall conclusions, there are specific insights from the French study that we see confirmed in our work. For example, Vieux et al. found that empty-calorie foods lower an overall dietary score, as well as total dietary GHGE. Similar findings were seen with our HEI subscore analysis of the refined grain and empty-calorie food groups, where the low-GHGE group actually ate more of these foods, scoring lower on these measures. Low-GHGE diets in our sample were not only higher in sugars, but also lower in several nutrients of public health concern, such as iron and calcium, another parallel to the Vieux et al. study, and other studies reviewed recently (39).

We focused on comparisons between the lowest and highest GHGE quintile diets because of the dramatic differences in emissions between these groups as well as for ease of exposition. When comparing diets across all 5 quintiles, there were a number of linear as well as nonlinear trends in intakes (see Supplemental Tables 1–3). Diets are complex with many ingredients that each influence nutritional quality and environmental impacts. This explains the nuanced relationship we observed between these outcomes at the diet level. It also implies that modifications can be made to existing diets to improve nutritional quality, reduce environmental impact, or both.

Women, on average, consume less energy than men, so a lower carbon footprint for women's diets would not be surprising. This has been observed in Germany (35), Australia (18), the United Kingdom (40), and Sweden (17). We see this in our data as well, even when standardized for energy intake, as diets in the lowest GHGE quintile group are more likely to be consumed by women, whereas the reverse is true for the highest quintile diets. African-Americans and younger individuals (18–29) were also more likely to have diets in the lowest quintile group.

Our results also point to associations between dietary GHGE and knowledge and concern for diet and nutrition. Use of food labels, being aware of dietary guidance (in the form of the Food Guide Pyramid or MyPyramid), or having tried dietary guidance is associated with lower dietary GHGE, even in multivariable models that control for basic demographic characteristics. Finally, we found a strong association between vegetarians and low-GHGE diets, which is not surprising given the high impacts of meats and similar results from previous research on this topic (13, 41, 42).

There are several strengths to our work including the matching of nationally representative self-selected diets in the United States to environmental impact data, the first of its kind for this country. Our environmental impact data come from a comprehensive review of LCA studies, including far more datapoints than typically used in this work. Finally, by connecting to the NHANES data, we are able to examine demographic, dietary, and behavioral characteristics of those consuming these diets.

Nonetheless, there are several limitations to this work. First, our work is based on 1-d dietary recalls, not usual intakes of individuals. However, the set of diets we observed are representative of those self-selected on a given day in the United States. After completing the 24-h recall, NHANES asks respondents if the amount of food eaten on the reporting day was much more than usual, usual, or much less than usual. As a check, we repeated our analyses, selecting only those diets that were reported as “usual” by the respondents. None of the significant findings we reported here were changed by this analysis. A second limitation is that our study only assessed GHGE. Additional environmental impacts, such as water and land use, which we plan to assess in the future, would allow us to make broader conclusions about our study.

A third limitation of our work is that we used only cradle-to-farm gate impacts for most linkages to NHANES data, and thereby underestimated impacts of the total diet. However, this approach was necessary, in part, because of the LCA literature, which more consistently reports these impacts, and in part because NHANES data do not include information enabling assessment of post-farm gate impacts. Using an alternative macrolevel approach, we estimated that processing and packaging would increase the average GHGE impact by ∼27% and transportation would add another ∼5% (19). Although the overall impacts of diets reported here are underestimates, there is no reason to believe that there are systematic differences that would affect the nutritional quality results presented.

There is significant interest among Americans about climate change and what can be done individually to mitigate human impacts (43). Given the pressing nature of global climate change and the food system's contribution to it, additional attention to dietary guidance that incorporates environmental impacts is clearly warranted. Although federal dietary guidance did not include mention of sustainability in the last round of the DGA (26), subsequent editions of these Guidelines will have significantly more research available to provide such guidance. Our work provides a baseline of the carbon footprint of US diets at the end of the first decade of the 21st century. Although we found that the relationship between dietary quality and environmental impact is nuanced, it is clear from our work that acceptable diets can be crafted that both reduce GHGE and improve overall nutritional quality.

## Supplementary Material

nqy327_Supplemental_File

## Notes

Supported by the Wellcome Trust (grant 106854/Z/15/Z).

Supplemental Tables 1–3 are available from the “Supplementary data” link in the online posting of the article and from the same link in the online table of contents at https://academic.oup.com/ajcn/.

Abbreviations used: CO_2_-eq, carbon dioxide equivalent; cup eq, cup equivalent; dataFIELD, database of Food Impacts on the Environment for Linking to Diets; DGA, Dietary Guidelines for Americans; FCID, Food Commodity Intake Database; GHGE, greenhouse gas emissions; HEI, Healthy Eating Index 2010; high-GHGE diet, diets in the top quintile of the distribution of GHGE/1000 kcal, which was calculated for each individual 1-d diet; LCA, life cycle assessment; low-GHGE diet, diets in the bottom quintile of the distribution of GHGE/1000 kcal, which was calculated for each individual 1-d diet; NHANES, National Health and Nutrition Examination Survey; USDA, US Department of Agriculture.
