# Supplementary material for: Carbon footprint of self-selected US diets: nutritional, demographic, and behavioral correlates
Source: Am J Clin Nutr. 2019 Jan 29;109(3):526–34. doi: 10.1093/ajcn/nqy327 (PMC6408204; doi:10.1093/ajcn/nqy327)
Supplement: nqy327_Supplemental_File [file nqy327_supplemental_file.docx]

**Supplemental Table 1**

**Nutrient intakes per 1000 kcal by dietary GHGE quintiles, Adults 18+ years, NHANES 2005-2010^1^**

|  | Lowest GHGE Quintile Diet^2^  (n=3,545) | Quintile 2 Diet^2^  (n=3,341) | Quintile 3 Diet^2^  (n=3,283) | Quintile 4 Diet^2^  (n=3,328) | Highest GHGE Quintile Diet^2^  (n=3,303) | P^3^ | LT^3^ | QT^3^ |
| --- | --- | --- | --- | --- | --- | --- | --- | --- |
| GHGE (kg CO_2_ eq) per 1000 kcal | 0.90 ± 0.00 | 1.31 ± 0.00 | 1.74 ± 0.00 | 2.55 ± 0.01 | 4.54 ± 0.03 | – | – | – |
| GHGE/1000 kcal, quintile % of total | 8.2% | 11.8% | 15.8% | 23.1% | 41.1% | – | – | – |
| Dietary fiber, g | 8.77 ± 0.16 | 8.27 ± 0.11 | 8.09 ± 0.12 | 7.64 ± 0.09 | 7.11 ± 0.11 | <0.001 | 0.000 | 0.434 |
| Vitamin A, µg RAE | 269.21 ± 7.97 | 337.64 ± 6.2 | 349.16 ± 8.29 | 302.18 ± 6.81 | 304.80 ± 9.96 | <0.001 | 0.195 | <0.001 |
| Vitamin C, mg | 40.10 ± 1.28 | 46.79 ± 1.53 | 48.23 ± 1.56 | 43.73 ± 1.65 | 43.03 ± 1.52 | <0.001 | 0.526 | <0.001 |
| Vitamin D (D2 + D3), µg | 1.75 ± 0.05 | 2.6 ± 0.09 | 2.72 ± 0.08 | 2.43 ± 0.07 | 2.11 ± 0.06 | <0.001 | 0.004 | <0.001 |
| Vitamin E as alpha-tocopherol, mg | 3.99 ± 0.08 | 3.79 ± 0.07 | 3.69 ± 0.06 | 3.45 ± 0.08 | 3.38 ± 0.1 | <0.001 | <0.001 | 0.658 |
| Total choline, mg | 119.72 ± 1.27 | 149.66 ± 1.7 | 168.09 ± 1.94 | 161.64 ± 1.66 | 192.99 ± 1.81 | <0.001 | <0.001 | 0.002 |
| Iron, mg | 7.44 ± 0.11 | 7.47 ± 0.09 | 7.33 ± 0.09 | 7.45 ± 0.08 | 7.99 ± 0.1 | <0.001 | <0.001 | 0.006 |
| Calcium, mg | 393.67 ± 5.24 | 491.01 ± 7.48 | 518.08 ± 6.69 | 482.91 ± 5.13 | 457.34 ± 5.71 | <0.001 | <0.001 | 0.000 |
| Magnesium, mg | 145.21 ± 1.92 | 149.03 ± 1.47 | 151.18 ± 1.49 | 143.4 ± 1.17 | 145.79 ± 1.6 | 0.009 | 0.341 | 0.031 |
| Potassium, mg | 1165.89 ± 13.16 | 1320.59 ± 12.01 | 1401.2 ± 14.42 | 1345.15 ± 11.11 | 1421.55 ± 16.04 | <0.001 | <0.001 | <0.001 |
| Sodium, mg | 1510.42 ± 12.95 | 1644.95 ± 16.3 | 1714.48 ± 13.03 | 1731.2 ± 12.65 | 1774.93 ± 15.96 | <0.001 | <0.001 | <0.001 |
| Total saturated fatty acids, g | 9.85 ± 0.09 | 11.89 ± 0.1 | 12.82 ± 0.09 | 13.15 ± 0.12 | 13.46 ± 0.09 | <0.001 | <0.001 | <0.001 |

^1^Values are mean ± SE. GHGE stands for greenhouse gas emissions.

^2^Lowest GHGE Quintile diets are those in the lowest quintile of the distribution of diets ranked by GHGE (kg CO_2_ equivalents) per 1000 kcal per day. Highest GHGE Quintile diets are those in the highest quintile of this distribution. Quintile 2, 3, and 4 Diets are those in the 2nd, 3rd, and 4th quintiles of this distribution.

^3^P values are for F-tests on linear regression models, which tested the overall relationship between nutrient intake and GHGE quintile groups. LT is the significance level for the linear trend test. QT is the significance level for the quadratic trend test. Statistical tests were not run on the GHGE continuous variable (first 2 rows), since the quintile groups were based on it.

**Supplemental Table 2**

**Food group intakes by dietary GHGE quintiles, Adults 18+ years, NHANES 2005-2010^1^**

|  | Unit^2^ | Lowest GHGE Quintile Diet^3^  (n=3,545) | Quintile 2 Diet^3^  (n=3,341) | Quintile 3 Diet^3^  (n=3,283) | Quintile 4 Diet^3^  (n=3,328) | Highest GHGE Quintile Diet^3^  (n=3,303) | P^4^ | LT^4^ | QT^4^ |
| --- | --- | --- | --- | --- | --- | --- | --- | --- | --- |
| Total fruit & vegetables^5^ | cup eq/1000 kcal | 1.19 ± 0.03 | 1.36 ± 0.03 | 1.43 ± 0.03 | 1.29 ± 0.02 | 1.30 ± 0.03 | <0.001 | 0.102 | <0.001 |
| Fruit | cup eq/1000 kcal | 0.49 ± 0.02 | 0.57 ± 0.02 | 0.6 ± 0.02 | 0.49 ± 0.02 | 0.46 ± 0.02 | <0.001 | 0.018 | <0.001 |
| Vegetables^5^ | cup eq/1000 kcal | 0.71 ± 0.02 | 0.78 ± 0.02 | 0.83 ± 0.02 | 0.8 ± 0.02 | 0.84 ± 0.02 | <0.001 | <0.001 | 0.033 |
| Total grains | oz eq/1000 kcal | 3.60 ± 0.05 | 3.26 ± 0.04 | 3 ± 0.03 | 2.97 ± 0.03 | 2.63 ± 0.03 | <0.001 | <0.001 | 0.097 |
| Whole grains | oz eq/1000 kcal | 0.51 ± 0.02 | 0.45 ± 0.02 | 0.39 ± 0.02 | 0.32 ± 0.01 | 0.28 ± 0.01 | <0.001 | <0.001 | 0.608 |
| Refined grains | oz eq/1000 kcal | 3.09 ± 0.05 | 2.82 ± 0.04 | 2.61 ± 0.03 | 2.65 ± 0.03 | 2.35 ± 0.03 | <0.001 | <0.001 | 0.198 |
| Protein foods: total^6^ | oz eq/1000 kcal | 2.37 ± 0.04 | 2.77 ± 0.05 | 3.14 ± 0.05 | 3.19 ± 0.05 | 4.17 ± 0.04 | <0.001 | <0.001 | <0.001 |
| Animal protein foods | oz eq/1000 kcal | 1.57 ± 0.03 | 2.23 ± 0.05 | 2.67 ± 0.04 | 2.74 ± 0.04 | 3.79 ± 0.04 | <0.001 | <0.001 | 0.006 |
| Meat | oz eq/1000 kcal | 0.11 ± 0.01 | 0.21 ± 0.01 | 0.47 ± 0.02 | 0.96 ± 0.02 | 2.26 ± 0.04 | <0.001 | <0.001 | <0.001 |
| Poultry | oz eq/1000 kcal | 0.85 ± 0.03 | 1.02 ± 0.04 | 0.94 ± 0.05 | 0.57 ± 0.03 | 0.33 ± 0.02 | <0.001 | <0.001 | <0.001 |
| Seafood | oz eq/1000 kcal | 0.21 ± 0.01 | 0.3 ± 0.02 | 0.33 ± 0.02 | 0.3 ± 0.03 | 0.41 ± 0.03 | <0.001 | <0.001 | 0.741 |
| Plant protein foods^7^ | oz eq/1000 kcal | 0.81 ± 0.04 | 0.54 ± 0.02 | 0.47 ± 0.02 | 0.45 ± 0.02 | 0.37 ± 0.02 | <0.001 | <0.001 | <0.001 |
| Total dairy | cup eq/1000 kcal | 0.54 ± 0.01 | 0.84 ± 0.02 | 0.93 ± 0.02 | 0.84 ± 0.02 | 0.72 ± 0.02 | <0.001 | <0.001 | <0.001 |
| Oils | g/1000 kcal | 12.86 ± 0.23 | 10.7 ± 0.22 | 9.87 ± 0.15 | 9.68 ± 0.2 | 8.40 ± 0.14 | <0.001 | <0.001 | 0.002 |
| Solid fats | g/1000 kcal | 14.67 ± 0.23 | 17.62 ± 0.21 | 18.52 ± 0.19 | 19.16 ± 0.24 | 19.02 ± 0.16 | <0.001 | <0.001 | <0.001 |
| Added sugars | tsp eq/1000 kcal | 10.46 ± 0.25 | 8.36 ± 0.18 | 7.82 ± 0.14 | 8.19 ± 0.16 | 7.43 ± 0.15 | <0.001 | <0.001 | <0.001 |

^1^Values are mean ± SE. GHGE stands for greenhouse gas emissions.

^2^Units for food groups were developed by USDA in common units and on an equivalent basis to create nutritional homogeneity in groups that have foods with diverse water concentrations (e.g. juice, fruit, or dried fruit). Cup eq/1000 kcal refers to cup equivalents per 1000 kilocalories. For example, 1 cup equivalent of dairy is either 1 cup (245 g) of milk, yogurt, or fortified soy milk, ~1.5 ounces of natural cheese, or ~2 ounces of processed cheese. Oz eq refers to ounce equivalents and tsp eq refers to teaspoon equivalents. See (27) for additional details.

^3^Lowest GHGE Quintile diets are those in the lowest quintile of the distribution of diets ranked by GHGE (kg CO_2_ equivalents) per 1000 kcal per day. Highest GHGE Quintile diets are those in the highest quintile of this distribution. Quintile 2, 3, and 4 Diets are those in the 2nd, 3rd, and 4th quintiles of this distribution.

^4^P values are for F-tests on linear regression models, which tested the overall relationship between nutrient intake and GHGE quintile groups. LT is the significance level for the linear trend test. QT is the significance level for the quadratic trend test.

^5^Vegetable totals do not include legumes.

^6^The total protein foods group is a sum of animal and plant protein foods.

^7^The plant protein foods group includes all legumes, soybeans, nuts, and seeds.

**Supplemental Table 3**

**Healthy Eating Index component and total scores by dietary GHGE quintiles, Adults 18+ years in the 2005-2010 NHANES^1^**

| Healthy Eating Index Component | Maximum score | Lowest GHGE Quintile Diet^2^  (n=3,545) | Quintile 2 Diet^2^  (n=3,341) | Quintile 3 Diet^2^  (n=3,283) | Quintile 4 Diet^2^  (n=3,328) | Highest GHGE Quintile Diet^2^  (n=3,303) | P^3^ | LT^3^ | QT^3^ |
| --- | --- | --- | --- | --- | --- | --- | --- | --- | --- |
| Total Fruit | 5 | 2.04 ± 0.07 | 2.40 ± 0.06 | 2.33 ± 0.06 | 2.06 ± 0.05 | 1.91 ± 0.05 | <0.001 | 0.003 | <0.001 |
| Whole Fruit | 5 | 2.06 ± 0.08 | 2.31 ± 0.05 | 2.21 ± 0.07 | 2.01 ± 0.06 | 1.77 ± 0.05 | <0.001 | <0.001 | <0.001 |
| Total Vegetables | 5 | 2.80 ± 0.05 | 2.94 ± 0.05 | 3.08 ± 0.04 | 3.07 ± 0.04 | 3.18 ± 0.05 | <0.001 | <0.001 | 0.200 |
| Greens and Beans | 5 | 1.14 ± 0.05 | 1.21 ± 0.05 | 1.27 ± 0.05 | 1.23 ± 0.06 | 1.19 ± 0.06 | 0.305 | 0.494 | 0.041 |
| Whole Grains | 10 | 2.74 ± 0.09 | 2.62 ± 0.09 | 2.34 ± 0.08 | 1.99 ± 0.07 | 1.77 ± 0.07 | <0.001 | <0.001 | 0.254 |
| Dairy | 10 | 4.03 ± 0.08 | 5.83 ± 0.10 | 6.02 ± 0.09 | 5.51 ± 0.08 | 4.83 ± 0.10 | <0.001 | <0.001 | <0.001 |
| Total Protein Foods | 5 | 3.56 ± 0.04 | 3.96 ± 0.04 | 4.19 ± 0.02 | 4.41 ± 0.02 | 4.87 ± 0.01 | <0.001 | <0.001 | 0.255 |
| Seafood and Plant Proteins | 5 | 2.38 ± 0.06 | 2.08 ± 0.06 | 1.96 ± 0.05 | 1.85 ± 0.06 | 1.60 ± 0.06 | <0.001 | <0.001 | 0.493 |
| Fatty Acids | 10 | 6.91 ± 0.08 | 5.15 ± 0.08 | 4.33 ± 0.09 | 4.11 ± 0.10 | 3.59 ± 0.07 | <0.001 | <0.001 | <0.001 |
| Refined Grains^4^ | 10 | 5.22 ± 0.12 | 5.81 ± 0.10 | 6.35 ± 0.07 | 6.27 ± 0.09 | 7.05 ± 0.08 | <0.001 | <0.001 | 0.537 |
| Sodium^4^ | 10 | 5.57 ± 0.09 | 4.68 ± 0.10 | 4.13 ± 0.09 | 4.07 ± 0.09 | 3.99 ± 0.10 | <0.001 | <0.001 | <0.001 |
| Empty Calories^4, 5^ | 20 | 11.81 ± 0.23 | 12.23 ± 0.21 | 12.26 ± 0.17 | 11.64 ± 0.18 | 12.26 ± 0.15 | 0.006 | 0.558 | 0.669 |
| Total HEI^6^ Score | 100 | 50.25 ± 0.57 | 51.22 ± 0.53 | 50.46 ± 0.42 | 48.22 ± 0.39 | 48.00 ± 0.42 | <0.001 | <0.001 | 0.012 |

^1^Values are mean ± SE. GHGE stands for greenhouse gas emissions.

^2^Lowest GHGE Quintile diets are those in the lowest quintile of the distribution of diets ranked by GHGE (kg CO_2_ equivalents) per 1000 kcal per day. Highest GHGE Quintile diets are those in the highest quintile of this distribution. Quintile 2, 3, and 4 Diets are those in the 2nd, 3rd, and 4th quintiles of this distribution.

^3^P values are for F-tests on linear regression models, which tested the overall relationship between nutrient intake and GHGE quintile groups. LT is the significance level for the linear trend test. QT is the significance level for the quadratic trend test.

^4^Higher component scores are considered beneficial. Thus, for refined grains, sodium, and empty calories, higher scores indicate diets that contain less of these items.

^5^Calories from solid fats, added sugars, and alcohol. For alcohol, intakes ≤ 13 grams/1000kcal do not influence scoring.

^6^HEI is the Healthy Eating Index, an overall index of diet quality based on the Dietary Guidelines for Americans. The 2010 version was used for this analysis (30).
